# Symptomatic periesophageal vagal nerve injury by different energy sources during atrial fibrillation ablation

**DOI:** 10.3389/fcvm.2023.1278603

**Published:** 2023-10-30

**Authors:** Shinsuke Miyazaki, Atsushi Kobori, Hikari Jo, Takehiko Keida, Kazuyasu Yoshitani, Moe Mukai, Yuichiro Sagawa, Tetsuya Asakawa, Eiji Sato, Kazuya Yamao, Tomoki Horie, Mamoru Manita, Hidehira Fukaya, Hidemori Hayashi, Kojiro Tanimoto, Tadateru Iwayama, Suguru Chiba, Akinori Sato, Yukio Sekiguchi, Kenta Sugiura, Shinsuke Iwai, Yuhei Isonaga, Naoyuki Miwa, Nobutaka Kato, Osamu Inaba, Takayoshi Hirota, Yasutoshi Nagata, Yuichi Ono, Hitoshi Hachiya, Yasuteru Yamauchi, Masahiko Goya, Junichi Nitta, Hiroshi Tada, Tetsuo Sasano

**Affiliations:** ^1^ Department of Cardiovascular Medicine, Tokyo Medical and Dental University, Tokyo, Japan; ^2^ Department of Cardiovascular Medicine, Kobe City Medical Center General Hospital, Hyogo, Japan; ^3^ Department of Cardiology, National Hospital Organization Higashi-Hiroshima Medical Center, Hiroshima, Japan; ^4^ Department of Cardiology, Edogawa Hospital, Tokyo, Japan; ^5^ Department of Cardiology, Hyogo Prefectural Amagasaki General Medical Center, Hyogo, Japan; ^6^ Department of Cardiovascular Medicine, Faculty of Medical Sciences, University of Fukui, Fukui, Japan; ^7^ Department of Cardiology, Japanese Red Cross Yokohama City Bay Hospital, Kanagawa, Japan; ^8^ Department of Cardiology, Yamanashi Kosei Hospital, Yamanashi, Japan; ^9^ Department of Cardiovascular Medicine, Sendai City Hospital, Miyagi, Japan; ^10^ Department of Cardiology, Ome Municipal General Hospital, Tokyo, Japan; ^11^ Department of Cardiology, Japanese Red Cross Musashino Hospital, Tokyo, Japan; ^12^ Department of Cardiology, Naha City Hospital, Okinawa, Japan; ^13^ Department of Cardiovascular Medicine, Kitasato University School of Medicine, Kanagawa, Japan; ^14^ Department of Cardiovascular Biology and Medicine, Juntendo University, Tokyo, Japan; ^15^ Department of Cardiology, National Hospital Organization Tokyo Medical Center, Tokyo, Japan; ^16^ Department of Cardiology, Okitama Public General Hospital, Yamagata, Japan; ^17^ Department of Cardiology, Urasoe General Hospital, Okinawa, Japan; ^18^ Cardiovascular Center, Tachikawa General Hospital, Niigata, Japan; ^19^ Department of Cardiology, Sakakibara Heart Institute, Tokyo, Japan; ^20^ Department of Cardiology and Geriatrics, Kochi University, Kochi, Japan; ^21^ Department of Cardiology, Hiratsuka Kyosai Hospital, Kanagawa, Japan; ^22^ Department of Cardiology, Japanese Red Cross Saitama Hospital, Saitama, Japan; ^23^ Cardiovascular Center, Tsuchiura Kyodo Hospital, Ibaraki, Japan

**Keywords:** complication, gastric hypomotility, vagal nerve injury, pulmonary vein isolation, atrial fibrillation, catheter ablation

## Abstract

**Background:**

Symptomatic gastric hypomotility (SGH) is a rare but major complication of atrial fibrillation (AF) ablation, but data on this are scarce.

**Objective:**

We compared the clinical course of SGH occurring with different energy sources.

**Methods:**

This multicenter study retrospectively collected the characteristics and clinical outcomes of patients with SGH after AF ablation.

**Results:**

The data of 93 patients (67.0 ± 11.2 years, 68 men, 52 paroxysmal AF) with SGH after AF ablation were collected from 23 cardiovascular centers. Left atrial (LA) ablation sets included pulmonary vein isolation (PVI) alone, a PVI plus a roof-line, and an LA posterior wall isolation in 42 (45.2%), 11 (11.8%), and 40 (43.0%) patients, respectively. LA ablation was performed by radiofrequency ablation, cryoballoon ablation, or both in 38 (40.8%), 38 (40.8%), and 17 (18.3%) patients, respectively. SGH diagnoses were confirmed at 2 (1–4) days post-procedure, and 28 (30.1%) patients required re-hospitalizations. Fasting was required in 81 (92.0%) patients for 4 (2.5–5) days; the total hospitalization duration was 11 [7–19.8] days. After conservative treatment, symptoms disappeared in 22.3% of patients at 1 month, 48.9% at 2 months, 57.6% at 3 months, 84.6% at 6 months, and 89.7% at 12 months, however, one patient required surgery after radiofrequency ablation. Symptoms persisted for >1-year post-procedure in 7 patients. The outcomes were similar regardless of the energy source and LA lesion set.

**Conclusions:**

The clinical course of SGH was similar regardless of the energy source. The diagnosis was often delayed, and most recovered within 6 months, yet could persist for over 1 year in 10%.

## Introduction

Catheter ablation of atrial fibrillation (AF) has become a widely accepted treatment strategy, and pulmonary vein isolation (PVI) is the cornerstone (1). Additional left atrial (LA) ablation is often performed in patients with persistent AF to overcome a relatively low rate of AF freedom after a PVI alone. However, ablation of the posterior LA using thermal energy could result in esophagus-related complications including periesophageal vagal nerve injury typically represented by gastric hypomotility (GH) (1, 2). Acute symptomatic GH after radiofrequency (RF) ablation (RFA) was initially reported in 2005 (2), followed by several case reports and small case series up to now (3–7). The incidence of symptomatic GH is generally very low, potentially because most instances of GH are of low severity and recover quickly. Indeed, the AF-GUT study clarified that an RF-PVI frequently results in transient asymptomatic functional impairment of the upper gastrointestinal system (8), and routine endoscopy post-RFA found that in 17% of asymptomatic GH cases (9). The exact incidence of this complication is unknown, however, we showed that the incidence of symptomatic GH after cryoballoon-based AF ablation was 0.23% (10). Due to the wide variation in the severity and very low incidence of this complication, real-world data is scarce. This study aimed to investigate the impact of the energy sources and LA lesion set on the clinical course of symptomatic GH after AF ablation.

## Methods

### Study design

Data of 93 patients who presented with symptomatic GH after AF ablation were retrospectively collected from a total of 23 cardiovascular centers using the database and medical records of each center. The data included the patients with GH secondary to CB ablation (CBA) in our previous study (10). The collected data included the patient characteristics and procedural, management, and follow-up data. AF was classified according to the latest guidelines (1). The study protocol was approved by the Tokyo Medical and Dental University and the institutional review board of each hospital. All patient information was anonymized, and the patients approved the use of their data for research purposes using an opt-out method. This study complied with the principles of the Declaration of Helsinki. The data that support the findings of this study are available from the corresponding author upon reasonable request.

### Ablation protocol

The intraprocedural management was performed according to the protocols of the individual centers. The procedure was performed under conscious sedation or deep sedation. Periprocedural anticoagulation therapy was performed according to the recommendations (1). With RFA, following a transseptal puncture, the ipsilateral pulmonary veins (PVs) were circumferentially ablated using an irrigated-tip catheter guided by 3-D mapping systems (CARTO, Biosense Webster, Diamond Bar, CA, USA or Ensite, St. Jude Medical, St. Paul, MN). In cases with esophageal temperature monitoring, the application was terminated if the temperature reached 39–41°C. With CBA, a freeze cycle of 180–240 s was applied following complete sealing of the PV, with a 28 mm cryoballoon (Arctic Front Advance or Artic Front Advance PRO, Medtronic Inc., Minneapolis, MN, USA, or POLARx, Boston Scientific, MA, USA). To avoid phrenic nerve injury, diaphragmatic electromyography was monitored during CBA of the right PVs. In cases with esophageal temperature monitoring, the application was terminated if the temperature reached 15–25°C. If the balloon temperature reached −55 to −60°C (−70°C in POLARx), or the electromyography amplitude significantly decreased, freezing was terminated. Adjunctive LA ablation was performed according to the operators' preference in a part of the sample.

### Definition of symptomatic gastric hypomotility

Patients were diagnosed with symptomatic GH if (1) they exhibited the following symptoms after AF ablation: acute onset of characteristic and prolonged symptoms of delayed gastric emptying, such as nausea, vomiting, postprandial fullness, bloating, constipation, or epigastric pain, and (2) findings of GH (gastric dilation and food retention) were objectively confirmed by abdominal x-ray, abdominal computed tomography, gastric endoscopy, and/or an upper gastrointestinal series (1). Recovery was defined as the complete disappearance of newly appearing gastroparesis-related symptoms.

### Follow-up

All patients were prescribed proton-pump inhibitors for ≥1-month post-procedure. The patients underwent continuous in-hospital electrocardiogram monitoring while hospitalized following the procedure. Usually, the patients were discharged 2 days after the procedure if no complications were observed. Subsequent follow-up was performed according to the recommendations of the latest guidelines (1) with a clinical interview, electrocardiograms, 24-h Holter monitoring, 7 days' Holter monitoring, and a long-term event recorder at each center. Recurrence was defined as any atrial tachyarrhythmias lasting longer than 30 s beyond the 3-month blanking period.

### Statistical analysis

Continuous data are expressed as the mean ± standard deviation for normally distributed variables or as the median (25th, 75th percentiles) for non-normally distributed variables, and were compared using a student's *t*-test or Mann-Whitney *U*-test, respectively. For a comparison of more than two group means, the one-way analysis of variance (ANOVA) was used. Categorical variables were compared using the Chi-square test or Fisher's exact test when the number of events was less than 5. A Kaplan-Meier analysis was used to determine the percentage of patients free from arrhythmia recurrence and GH-related symptoms. The differences in the GH-related symptoms were evaluated using the log-rank test. A Multivariate Cox regression model was used to determine the predictors of recovery of symptomatic GH, and the variables whose univariate analyses had a *p*-value <0.1 were included. Statistical significance was set at *P* < 0.05.

## Results

### Patient characteristics and the lesion set

Data from a total of 93 patients that presented with symptomatic GH were retrospectively collected from a total of 23 centers (Table 1). Among them, 38 patients were collected from 11,175 patients who underwent RFA at 6 centers that had at least 1 case with GH after RFA, and the remaining 55 patients were collected from 12,137 patients who underwent CBA at 21 centers that had at least 1 case with GH after CBA. The mean age was 67.0 ± 11.2 years, 68 (73.1%) patients were men, and 52 (55.9%) had paroxysmal AF. Among them, 42 (45.2%) patients underwent a PVI alone (PVI-group), 11 (11.8%) a PVI plus LA roof line ablation (Roof-group), and the remaining 40 (43.0%) an LA posterior wall isolation (LAPWI) (LAPWI-group) as an LA ablation (Table 2). The PVI-group was older and had a significantly higher prevalence of paroxysmal AF than the other groups, leading to a smaller LA size and higher left ventricular ejection fraction.

**Table 1:** Characteristics of the study population.

	*n* = 93
Age, years	67.0 ± 11.2
Male gender, *n* (%)	68 (73.1%)
Paroxysmal atrial fibrillation, *n* (%)	52 (55.9%)
Weight, kg	61.9 ± 11.0
Body mass index, kg/m^2^	23.5 ± 3.4
Left atrial diameter, mm	39.8 ± 8.0
Left ventricular ejection fraction, %	61.7 ± 12.0
Structural heart disease, *n* (%)	12 (12.9%)
Ischemic heart disease, *n* (%)	5 (5.4%)
Hypertrophic cardiomyopathy, *n* (%)	3 (3.2%)
Valvular heart disease, *n* (%)	4 (4.3%)
Others, *n* (%)	1 (1.1%)
CHADS_2_ score	1.3 ± 1.1
CHA_2_DS_2_VASc score	2.3 ± 1.5

Values are reported as the mean ± standard deviation or number of patients (%) unless otherwise noted. *n*: number.

**Table 2:** Patient characteristics in the PVI-group, roof-group, and LAPWI-group.

	PVI-group	Roof-group	LAPWI-group	*P*-value
	*n* = 42	*n* = 11	*n* = 40	
Age, years	69.9 ± 8.9	68.8 ± 11.8	63.4 ± 12.5	0.029
Male gender, *n* (%)	29 (69.1%)	8 (72.7%)	31 (77.5%)	0.688
Paroxysmal atrial fibrillation, *n* (%)	36 (85.7%)	2 (18.2%)	14 (35.0%)	<0.0001
Weight, kg	60.3 ± 9.9	61.5 ± 10.5	63.7 ± 12.1	0.375
Body mass index, kg/m^2^	23.2 ± 3.4	23.4 ± 4.0	23.8 ± 3.4	0.757
Left atrial diameter, mm	37.3 ± 6.4	42.5 ± 5.8	41.8 ± 9.3	0.019
Left ventricular ejection fraction, %	66.0 ± 6.5	59.5 ± 12.3	57.8 ± 15.1	0.008
Structural heart disease, *n* (%)	5 (13.1%)	3 (5.9%)	4 (15.8%)	0.307
CHADS_2_ score	1.1 ± 1.0	2.2 ± 1.3	1.1 ± 1.0	0.012
CHA_2_DS_2_VASc score	2.3 ± 1.4	3.2 ± 1.3	2.0 ± 1.5	0.047
RFA alone, *n* (%)	16 (38.1%)	3 (27.3%)	19 (47.5%)	
RFA plus CBA, *n* (%)	0 (0%)	2 (18.2%)	15 (37.5%)	
CBA alone, *n* (%)	26 (61.9%)	6 (54.5%)	6 (15.0%)	
Esophageal temperature monitoring, *n* (%)	30 (71.4%)	9 (81.8%)	34 (85.0%)	0.314

Values are reported as the mean ± standard deviation or number of patients (%) unless otherwise noted. CBA, cryoballoon ablation: LAPWI, left atrial posterior wall isolation: *n*, number; PVI, pulmonary vein isolation; RFA, radiofrequency ablation.

LA ablation was performed by RFA alone in 38 (40.8%) patients (RFA-group), a CBA alone in 38 (40.8%) (CBA-group), and a CBA plus RFA in the remaining 17 (18.3%) (CBA + RFA-group) (Table 3). The CBA-group was significantly older and had a greater body mass index than the RFA-group, leading to higher CHADS_2_ and CHA_2_DS_2_VASc scores. Among 42 PVI-group patients, a PVI was performed by RFA alone and CBA alone in 16 (38.1%) and 26 (61.9%) patients, respectively. Among 40 LAPWI-group patients, an LAPWI was performed by RFA alone and CBA alone in 19 (47.5%) and 6 (15.0%) patients, respectively (Table 2). Dexmedetomidine and propofol were used during the procedure for sedation in 33 (35.5%) and 56 (60.2%) patients, respectively. The esophageal temperature was monitored during the procedure in a total of 73 patients (78.5%) (Tables 1, 2). A PVI was performed by CBA in a total of 55 patients (all patients in the CBA-group and CBA + RFA-group).

**Table 3:** Patient characteristics in the RFA-group, CBA + RFA-group, and CBA-group.

	RFA-group	CBA + RFA-group	CBA-group	*P*-value
	*n* = 38	*n* = 17	*n* = 38	
Age, years	62.8 ± 11.6	66.7 ± 12.1	71.4 ± 8.9	0.003
Male gender, *n* (%)	29 (76.3%)	8 (47.1%)	31 (81.6%)	0.033
Paroxysmal atrial fibrillation, *n* (%)	19 (50.0%)	8 (47.1%)	25 (65.8%)	0.275
Weight, kg	60.3 ± 10.2	62.0 ± 11.0	63.4 ± 11.8	0.466
Body mass index, kg/m^2^	22.4 ± 3.1	24.6 ± 3.1	24.1 ± 3.7	0.031
Left atrial diameter, mm	40.1 ± 8.9	40.6 ± 9.5	39.2 ± 6.4	0.809
Left ventricular ejection fraction, %	60.2 ± 14.7	59.6 ± 14.4	64.2 ± 6.7	0.267
Structural heart disease, *n* (%)	5 (13.1%)	1 (5.9%)	6 (15.8%)	0.556
CHADS_2_ score	0.8 ± 1.1	1.6 ± 1.1	1.5 ± 1.1	0.009
CHA_2_DS_2_VASc score	1.6 ± 1.3	2.8 ± 1.7	2.7 ± 1.3	0.002
Esophageal temperature monitoring, *n* (%)	24 (63.2%)	15 (88.2%)	34 (89.5%)	0.012

Values are reported as the mean ± standard deviation or number of patients (%) unless otherwise noted. CBA, cryoballoon ablation: *n*, number; RFA, radiofrequency ablation.

### Diagnosis and management

All the patients took proton-pump inhibitors after the procedure. The patients were clinically diagnosed with GH at a median of 2 (1–4) (maximal 34) days after the procedure, and 40 patients (43.0%) required ≥3 days post-procedure for the diagnosis. Twenty-eight patients (30.1%) required re-hospitalization due to severe symptoms manifesting after discharge. All 93 patients exhibited typical symptoms including nausea, vomiting, and bloating. Among the 43 patients in whom weight data were available, 26 (60.5%) had a mean weight loss of 3 (2–4) (maximal 9) kg. In addition to an abdominal x-ray, 60 (64.5%), 36 (38.7%), and 12 patients (12.9%) underwent abdominal computed tomography, gastric endoscopy, and an upper gastrointestinal series, respectively. Abdominal computed tomography and gastric endoscopy were performed multiple times for an assessment in 16 (17.2%) and 16 patients (17.2%), respectively. The total hospitalization period (including the re-hospitalization period) was 11 (7–19.8) (maximal 80) days, and the period was similar between the energy sources (11 [6.5–20.2] in CBA-group, 10 [8–18.2] in CBA + RFA-group, and 12 [6.5–24] days in RFA-group, *p* = 0.98) and the LA lesion set (12 [7–21.2] in PVI-group, 13.5 [8.7–19.7] in Roof-group, 8.5 [4.7–13.5] days in LAPWI-group, *p* = 0.44).

A fast was required in 81 (92.0%) patients for 4 (2.5–5) (maximal 30) days among the 88 patients in whom the data were available. A low-residue diet was required in 68 (97.1%) of 70 patients in whom the data were available for 10.0 (6–18.7) (maximal 180) days. A gastric tube was inserted during the acute phase in at least 27 patients for 4 (1.5–6) days. In addition to acid suppressants (proton-pump inhibitors, etc.), mosapride was the most commonly used medication for gastroparesis for 48 (27–150) days in 77 (82.8%) patients, followed by antiemetics in 45 (48.4%) patients for 7 (4–30) days, erythromycin in 16 (17.2%) patients for 4 (2–14) days, Rikkunshito (Chinese herbal medicine) in 30 patients for 90 (43–150) days, Daikenchuto (Chinese herbal medicine) in 23 (24.7%) patients for 48.5 (16.5–127.5) days, panthenol in 27 (29.0%) patients for 7 (5–10) days, and acotiamide in 9 (9.7%) patients for 96 (42–210) days (Table 4).

**Table 4:** Medication for symptomatic gastric hypomotility.

Medication	
Acid suppressants	93 (100%)
Mosapride	77 (82.8%)
Antiemetics	45 (48.4%)
Erythromycin	16 (17.2%)
Rikkunshito	30 (32.2%)
Daikenchuto	23 (24.7%)
Panthenol	27 (29.0%)
Acotiamide	9 (9.7%)

### Clinical outcomes of Gh

**Figure 1:**
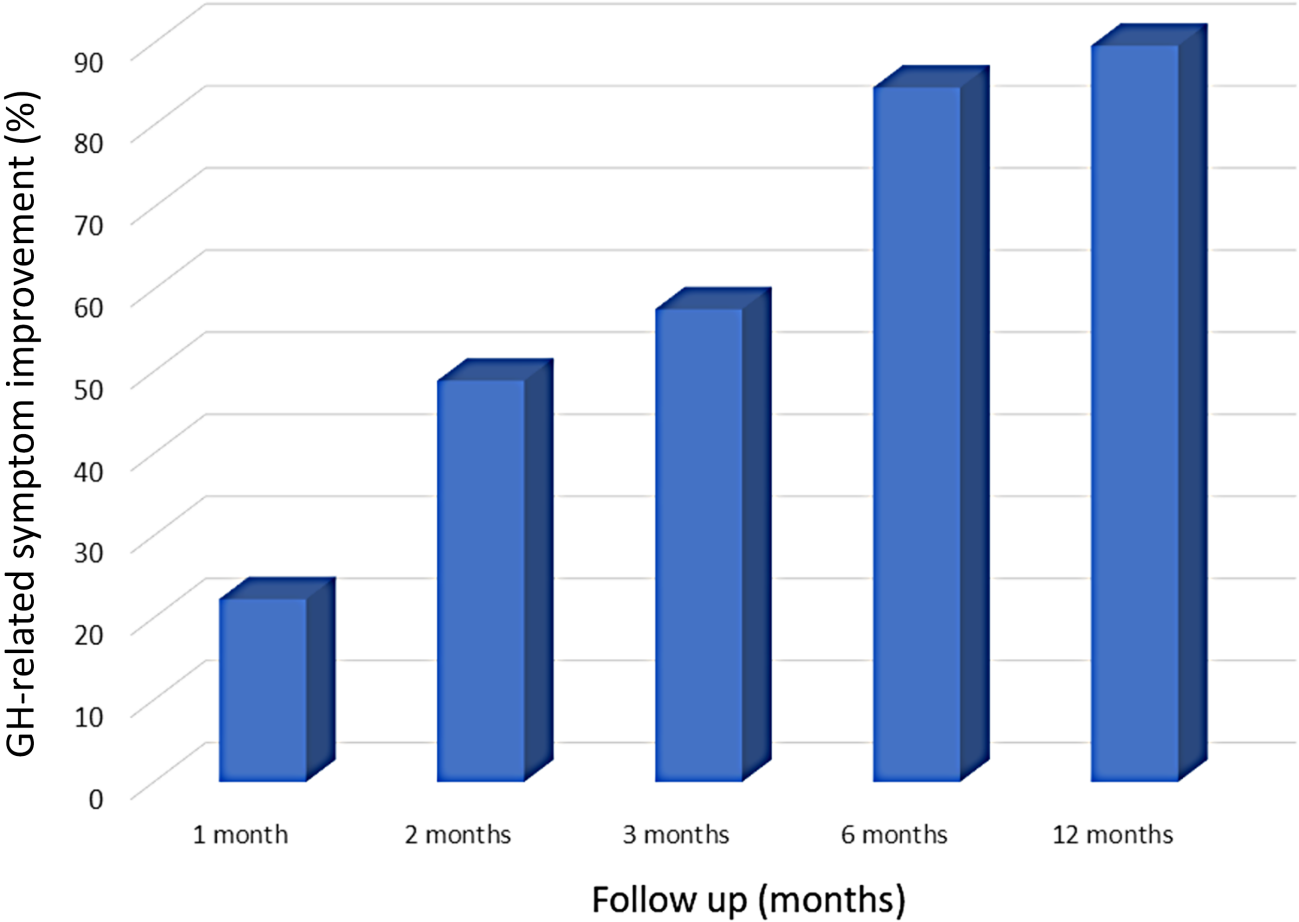
The GH-related symptom improvement after AF ablation. GH, gastric hypomotility.

During 24 (12–41.5) months of follow-up, symptoms disappeared in 22.3% of patients at 1 month, 48.9% at 2 months, 57.6% at 3 months, 84.6% at 6 months, and 89.7% at 12 months (Figure 1). In some patients, the postoperative follow-up period was still short. Symptoms remained at the last postoperative visit in 11 (11.8%) patients, at <3 months, and >1 year in 4 and 7 patients, respectively. Among the 7 patients with remaining symptoms >1 year postoperatively, 4, 3, 3, and 3 patients were in the PVI-group, LAPWI-group, RFA-group, and CBA-group, respectively. One of the patients who underwent a PVI alone with RFA required bypass surgery one month after the procedure due to a poor recovery of the gastric function despite medical therapy under continued hospitalization.

A univariate Cox regression analysis demonstrated that esophageal temperature monitoring was the sole significant factor associated with a longer recovery time of symptomatic GH (hazard ratio [HR] = 0.548, 95% confidence interval [CI] = 0.321–0.933; *p* = 0.027). A multivariate Cox regression analysis determined that esophageal temperature monitoring (HR = 0.548, 95% CI = 0.321–0.933; *p* = 0.027) was still a significant factor associated with a longer recovery time of symptomatic GH (Table 5). The recovery of GH-related symptoms was significantly faster in patients without esophageal temperature monitoring than those with (log-rank, *p* = 0.043). On the contrary, it was similar between the RFA-group and CBA-group (log-rank, *p* = 0.500), and between the PVI-group and LAPWI-group (log-rank, *p* = 0.893).

**Table 5:** Factors associated with the time course of the gastric hypomotility-related symptom improvement.

	Univariate			Multivariate		
	HR	*P*-value	95% CI	HR	*P*-value	95% CI
Age, years	1.009	0.443	0.986–1.032			
Male gender	0.977	0.928	0.587–1.624			
Paroxysmal atrial fibrillation	0.816	0.392	0.512–1.300			
Weight, kg	0.993	0.526	0.971–1.015			
Body mass index, kg/m^2^	0.979	0.539	0.914–1.048			
Left atrial diameter, mm	1.001	0.967	0.971–1.031			
CHADS_2_ score	1.208	0.077	0.980–1.488	1.245	0.046	1.004–1.544
CHA_2_DS_2_VASc score	1.118	0.155	0.959–1.305			
Esophageal temperature monitoring	0.586	0.046	0.346–0.992	0.548	0.027	0.321–0.933

CI, confidence interval; HR, hazard ratio.

## Discussion

To date, this was the largest study to analyze the characteristics and clinical outcomes of symptomatic GH after AF ablation in real-world clinical practice. We found that (1) the clinical course of symptomatic GH after RFA and CBA was similar, (2) the clinical course was also similar regardless of the LA lesion set, (3) 30% of the population required re-hospitalizations due to delays in symptom manifestation, and the total hospitalization period was a median of 11 days, (4) the symptoms disappeared in most of the patients within 6 months of conservative treatment; however, symptoms could persist for over 1 year in 10% of the population, and (5) esophageal temperature monitoring was associated with a longer recovery time.

### GH after RFA and CBA

Collateral damage could occur secondary to AF ablation regardless of the energy sources, yet the reported incidence differs between them. Phrenic nerve injury is observed more frequently with CBA than RFA, (11, 12) however, the recovery is faster with CBA. The reported incidence of atrioesophageal fistulae is higher with RFA than CBA. (1, 13) Though both RFA and CBA are a thermal ablation, these differences could be explained by the differences in the configuration of the ablation catheter and the mechanisms of lesion formation. (14, 15) On the contrary, the data of symptomatic GH has been limited due to the very low incidence and non-lethal complications despite impairing the patients' quality of life. The present study, for the first time, clarified that the outcomes of symptomatic GH were similar between RFA and CBA once it occurred, although the exact incidence was unknown from the present data. Interestingly, the reported incidence of asymptomatic endoscopy-detected GH (food retention in endoscopy) after CB-PVI (17%–28%) (16, 17) is relatively higher than RF-PVI (5.7%–18%). (17, 18) It is important to recognize that symptomatic GH is the tip of the iceberg of functional impairment of the upper gastrointestinal system and that most instances of GH are of low severity and recover quickly, irrespective of the energy sources.

### GH and the lesion set

In the present sample, the PVI-group was older and had a significantly higher prevalence of paroxysmal AF than the LAPWI-group. This might be because physicians preferred a PVI alone for paroxysmal AF and elderly patients and performed additional ablation for persistent AF considering the relatively lower AF freedom after a PVI alone. Some prior studies demonstrated that an LAPWI increased the risk of endoscopy-detected asymptomatic GH as compared to a PVI alone. (19, 20) However, the outcomes of GH were similar regardless of the LA lesion set once the vagal nerve injury exceeded the threshold of symptom appearance in the present study.

### Diagnosis, management, and outcomes

It is notable that ≥3 days were required to reach a diagnosis after the procedure in 40% of the sample because the symptoms were generally exacerbated by a full stomach. As a result, 30% of the sample required re-hospitalization after discharge. For treatment, 92% of the patients required a median of 4 days of fasting, followed by a median of 10 days of a low-residue diet. With this management, a median of an 11-day hospital stay was needed. As encouraged in the guidelines (1), most patients took mosapride and antiemetics. The symptoms completely recovered within 6 months in most of the patients; however, symptoms remained in a part of the sample despite these medications. In addition, one patient required surgical management. Clearly, symptomatic GH is an important major complication impairing the patient's quality of life.

Interestingly, esophageal temperature monitoring was associated with a longer recovery time of symptomatic GH in our sample. The feasibility of esophageal temperature monitoring is still controversial for AF ablation. (1) This is especially true for anticipating GH (21) because GH occurs due to injury to the vagal nerve network but not direct esophageal injury. Unfortunately, avoiding vagal nerve injury seems challenging, given the anatomic variability and lack of a modality for visualizing the vagal nerve. We assumed that the energy deliveries to the posterior LA were more carefully performed to avoid the potential risk of esophagus-related complications in patients without an esophageal temperature probe, which might explain the present study results. In the near future, it is expected that a new non-thermal energy source, pulsed-field ablation, will resolve this issue.

### Study limitations

First, detailed patient data and ablation strategies for patients without GH were not collected. In addition, the data were retrospectively collected from hospitals that had at least 1 case with symptomatic GH after AF ablation. Therefore, the exact incidence and predictors of GH could not be examined. Second, the ablation strategies and techniques might have differed at each center, and the amount of RFA was unknown. Third, scintigraphy and electrogastrography to evaluate the gastric function are unavailable in Japan. Therefore, the severity of GH could not be objectively assessed.

## Conclusions

The clinical course of symptomatic GH was similar regardless of the energy sources used and the LA lesion set created. The symptoms could appear with a delay and disappear within 6 months of conservative treatment in most populations; however, they could persist for more than 1 year in 10% of the population.

## Data Availability

The raw data supporting the conclusions of this article will be made available by the authors, without undue reservation.

## References

[1] Calkins H Hindricks G Cappato R Kim YH Saad EB Aguinaga L 2017 HRS/EHRA/ECAS/APHRS/SOLAECE expert consensus statement on catheter and surgical ablation of atrial fibrillation: executive summary. Heart Rhythm. (2017) 14:e445–94. 10.1016/j.hrthm.2017.07.009 31631881

[2] Shah D Dumonceau JM Burri H Sunthorn H Schroft A Gentil-Baron P Acute pyloric spasm and gastric hypomotility: an extracardiac adverse effect of percutaneous radiofrequency ablation for atrial fibrillation. J Am Coll Cardiol. (2005) 46:327–30. 10.1016/j.jacc.2005.04.030 16022963

[3] Bunch TJ Ellenbogen KA Packer DL Asirvatham SJ. Vagus nerve injury after posterior atrial radiofrequency ablation. Heart Rhythm. (2008) 5:1327–30. 10.1016/j.hrthm.2008.05.014 18774112

[4] Kuwahara T Takahashi A Takahashi Y Kobori A Miyazaki S Takei A Clinical characteristics and management of periesophageal vagal nerve injury complicating left atrial ablation of atrial fibrillation: lessons from eleven cases. J Cardiovasc Electrophysiol. (2013) 24:847–51. 10.1111/jce.12130 23551640

[5] Miyazaki S Taniguchi H Kusa S Komatsu Y Ichihara N Takagi T Factors associated with periesophageal vagal nerve injury after pulmonary vein antrum isolation. J Am Heart Assoc. (2014) 3:e001209. 10.1161/JAHA.114.001209 25249299 PMC4323793

[6] Aksu T Golcuk S Guler TE Yalin K Erden I. Gastroparesis as a complication of atrial fibrillation ablation. Am J Cardiol. (2015) 116:92–7. 10.1016/j.amjcard.2015.03.045 25933733

[7] Jacobs V May HT Crandall BG Ballantyne B Chisum B Johnson D Vagus nerve injury symptoms after catheter ablation for atrial fibrillation. Pacing Clin Electrophysiol. (2018) 41:389–95. 10.1111/pace.13304 29435991

[8] Lakkireddy D Reddy YM Atkins D Rajasingh J Kanmanthareddy A Olyaee M Effect of atrial fibrillation ablation on gastric motility: the atrial fibrillation gut study. Circ Arrhythm Electrophysiol. (2015) 8:531–6. 10.1161/CIRCEP.114.002508 25772541

[9] Knopp H Halm U Lamberts R Knigge I Zachäus M Sommer P Incidental and ablation-induced findings during upper gastrointestinal endoscopy in patients after ablation of atrial fibrillation: a retrospective study of 425 patients. Heart Rhythm. (2014) 11:574–8. 10.1016/j.hrthm.2014.01.010 24418167

[10] Miyazaki S Kobori A Jo H Keida T Yoshitani K Mukai M Symptomatic gastroparesis after cryoballoon-based atrial fibrillation ablation: results from a large multicenter registry. Circ Arrhythm Electrophysiol. (2023) 16(3):e011605. 10.1161/CIRCEP.122.011605 36745559

[11] Kuck KH Brugada J Fürnkranz A Metzner A Ouyang F Chun KR Cryoballoon or radiofrequency ablation for paroxysmal atrial fibrillation. N Engl J Med. (2016) 374:2235–45. 10.1056/NEJMoa1602014 27042964

[12] Schmidt M Dorwarth U Andresen D Brachmann J Kuck KH Kuniss M Cryoballoon versus RF ablation in paroxysmal atrial fibrillation: results from the German ablation registry. J Cardiovasc Electrophysiol. (2014) 25:1–7. 10.1111/jce.12267 24134539

[13] Piccini JP Braegelmann KM Simma S Koneru JN Ellenbogen KA. Risk of atrioesophageal fistula with cryoballoon ablation of atrial fibrillation. Heart Rhythm O2. (2020) 1:173–9. 10.1016/j.hroo.2020.05.007 34113871 PMC8183952

[14] Avitall B Kalinski A. Cryotherapy of cardiac arrhythmia: from basic science to the bedside. Heart Rhythm. (2015) 12:2195–203. 10.1016/j.hrthm.2015.05.034 26031374

[15] Goff RP Bersie SM Iaizzo PA. In vitro assessment of induced phrenic nerve cryothermal injury. Heart Rhythm. (2014) 11:1779–84. 10.1016/j.hrthm.2014.06.022 24952149

[16] Miyazaki S Nakamura H Taniguchi H Hachiya H Takagi T Igarashi M Gastric hypomotility after second-generation cryoballoon ablation-unrecognized silent nerve injury after cryoballoon ablation. Heart Rhythm. (2017) 14:670–7. 10.1016/j.hrthm.2017.01.028 28434448

[17] Oikawa J Fukaya H Wada T Kishihara J Sato T Matsuura G Esophagogastric complications in radiofrequency and cryoballoon catheter ablation of atrial fibrillation. J Cardiovasc Electrophysiol. (2022) 33:1160–6. 10.1111/jce.15518 35488745

[18] Yamasaki H Tada H Sekiguchi Y Igarashi M Arimoto T Machino T Prevalence and characteristics of asymptomatic excessive transmural injury after radiofrequency catheter ablation of atrial fibrillation. Heart Rhythm. (2011) 8:826–32. 10.1016/j.hrthm.2011.01.045 21315839

[19] Oikawa J Fukaya H Wada T Horiguchi A Kishihara J Satoh A Additional posterior wall isolation is associated with gastric hypomotility in catheter ablation of atrial fibrillation. Int J Cardiol. (2021) 326:103–8. 10.1016/j.ijcard.2020.10.069 33130261

[20] Yakabe D Fukuyama Y Araki M Nakamura T. Anatomical evaluation of the esophagus using computed tomography to predict acute gastroparesis following atrial fibrillation ablation. J Arrhythm. (2021) 37:1330–6. 10.1002/joa3.12625 34621432 PMC8485813

[21] Miyazaki S Nakamura H Taniguchi H Takagi T Iwasawa J Watanabe T Esophagus-related complications during second-generation cryoballoon ablation-insight from simultaneous esophageal temperature monitoring from 2 esophageal probes. J Cardiovasc Electrophysiol. (2016) 27:1038–44. 10.1111/jce.13015 27221011

